# Supplementary material for: CRISPR screens identify tumor‐promoting genes conferring melanoma cell plasticity and resistance
Source: EMBO Mol Med. 2021 Mar 16;13(5):e13466. doi: 10.15252/emmm.202013466 (PMC8103100; doi:10.15252/emmm.202013466)
Supplement: Supplementary file 1 — Appendix [file EMMM-13-e13466-s013.pdf]

APPENDIX

Gain-of-function CRISPR screens identify tumor-promoting genes conferring melanoma cell plasticity and resistance

Content:

Legends of Appendix Figures S1-S3.

Appendix Fig. S1-S3

Appendix Table S1 (Stats)

Supplementary Methods (CRISPR screen)

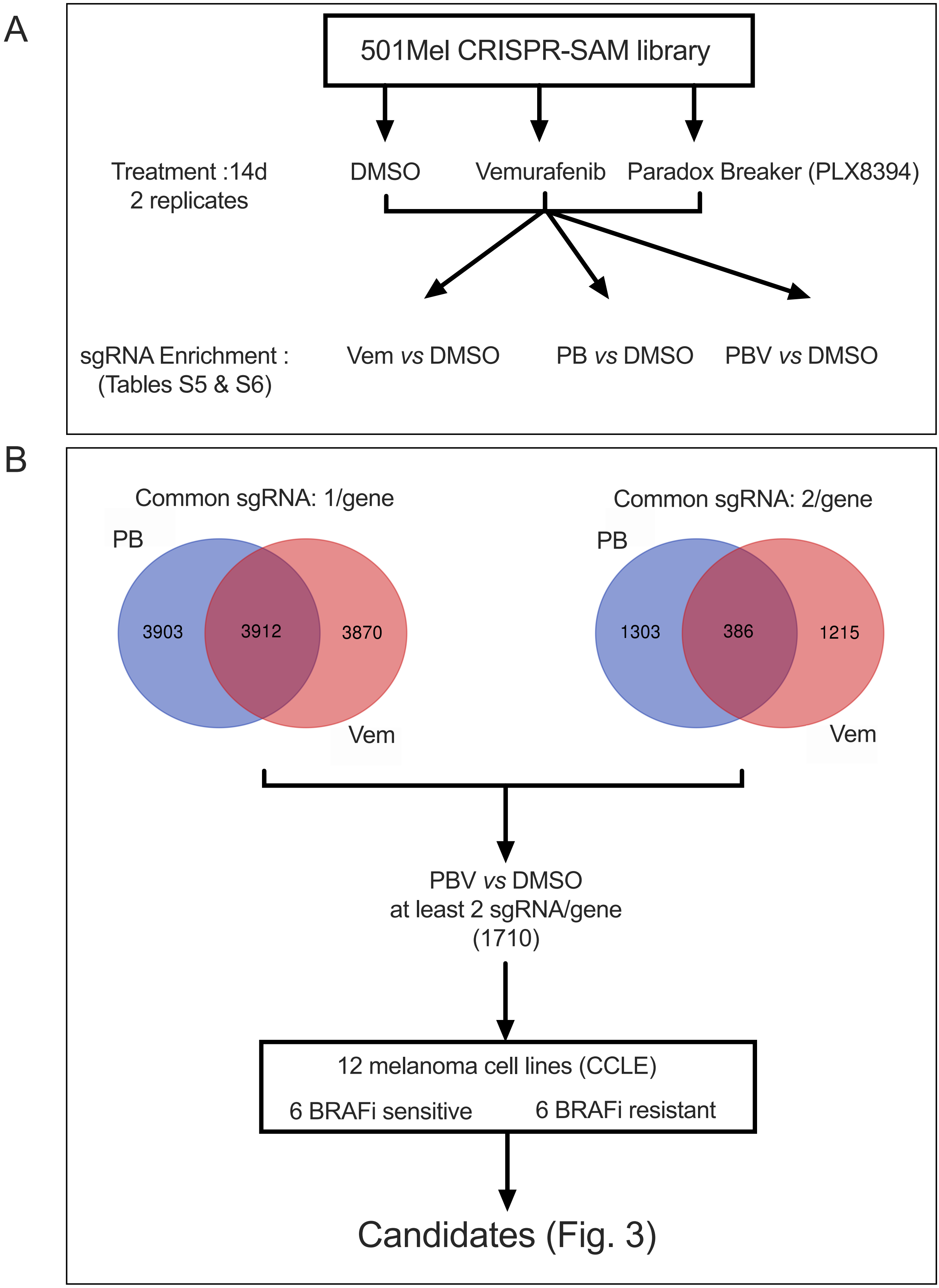

Appendix Figure S1.
sgRNAs enrichment in BRAFi-resistant cells

SMAD3 Immunostainings
PDX : MEL003, MEL006, MEL007, MEL037
T0 : drug naive
TRes : Dab+Tra resistant
Blue: DAPI
Green: SMAD3 (#9513)

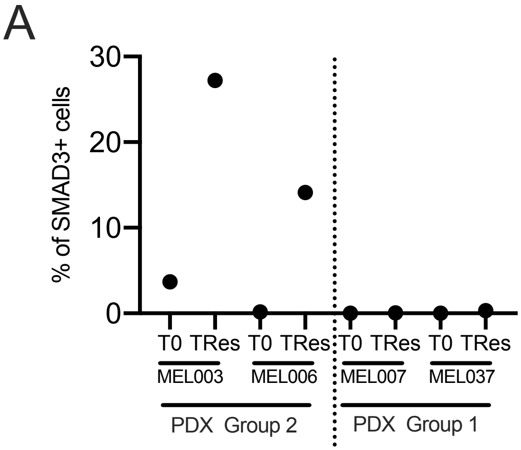

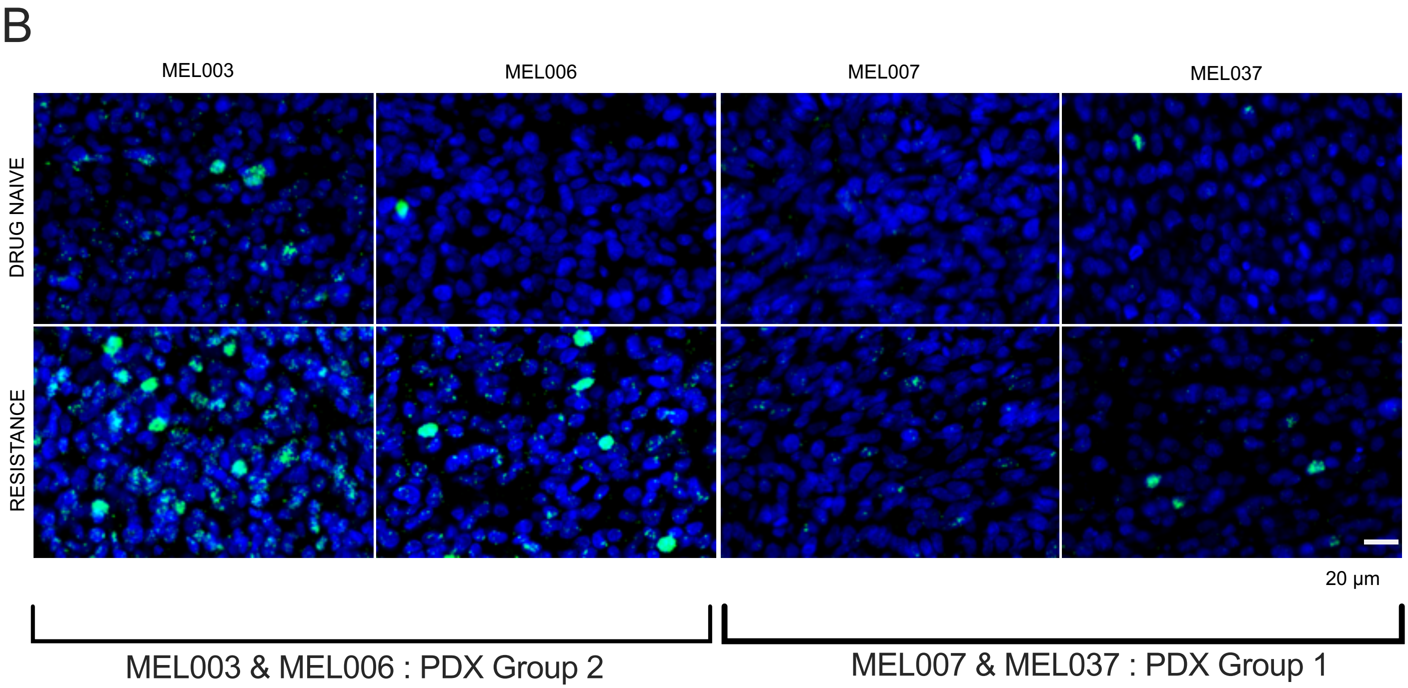

Appendix Figure S2.
SMAD3 immunostaining in PDX models

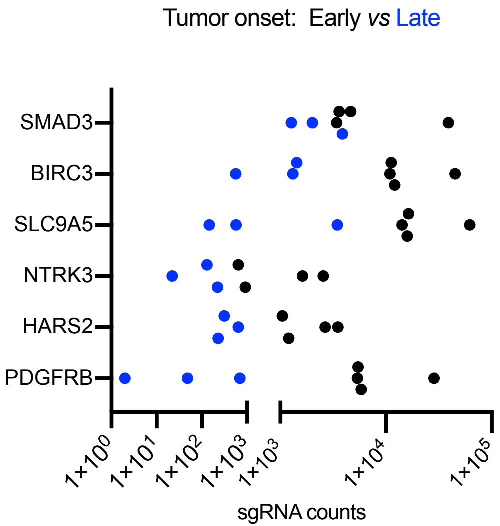

Appendix Figure S3.
Tumor growth from BRAFi-persister cells and tumor-promoting genes

Appendix Figure S1: sgRNAs enrichment in BRAFi-resistant cells

A-The CRISPR-SAM 501Mel cell library ($40x10^6$ cells) was treated for 14 days with BRAFi (2 µM), using either the BRAFi used in clinical practice (vemurafenib, (Vem)), the next generation inhibitor that is still under investigation in clinical trials (PLX8394, (PB)), or the solvent (dimethyl sulfoxide (DMSO)) as control. This procedure allows for the enrichment of sgRNAs (genes) conferring resistance. Median-normalized sgRNAs counts to adjust for the effect of library sizes and read count distributions of the CRISPR-SAM screens were assessed in 501Mel cell library (DMSO) and Vem- or PB-resistant cells. In each condition, sgRNA were sequenced from two independent experiments and reads were pooled before counting and normalization (Table EV5).

B-The two inhibitors were compared (1 or 2 sgRNA/gene) (Table EV6). Due to the partial overlapping, we decided to retain genes with at least two sgRNAs among the enriched sgRNAs present in BRAFi (PBV)-exposed cells (with a false discovery rate, FDR <0.05). Next, we confronted CRISPR-SAM candidates to gene expression data from six melanoma cell lines that were intrinsically highly resistant to BRAFi according to the Cancer Cell Lines Encyclopedia (CCLE) *versus* six sensitive cell lines. The goal was to identify BRAFi-resistant genes broadly found in BRAFi-resistant tumors and cell lines (Fig. 3).

Appendix Figure S2: SMAD3 immunostaining in PDX models

A-Percentage of SMAD3 positive cells in four BRAF-mutant PDX models exposed to BRAF/MEK inhibitors until resistance (To: drug naïve and TRes; tumour resistant to Dabrafenib+Trametinib treatment). The PDX models (all established from treatment naïve patients) have been recently characterized (Marin-Bejar *et al*, 2020). The PDXs MEL003 and MEL006 (group 2) initially respond to treatment, later develop resistance (after at least 60 days)

whereas drug responses were relatively modest and limited in time for MEL007 and MEL037 (group 1). Whereas group 1 reflected what is commonly referred to as intrinsic resistance, group 2 mimicked acquired resistance (group 2) (Marin-Bejar *et al*, 2020).

B- Representative images of SMAD3 immunostainings. Immunostainings show the emergence of SMAD3 positive cells upon MAPK-inhibition in DT-resistant lesions from the MEL003 and MEL006 PDXs (group 2).

Source data are available in Figure appendix S2 source data.

Appendix Figure S3: Tumor growth from BRAFi-persister cells and tumor-promoting genes

From Figure 4B, two groups of tumors emerging from BRAFi-persister cells (early and late, n=4 and 3 respectively) were made and the sgRNA enrichment was analyzed (early : Black dots and late : Blue dots). The sgRNAs targeting our best candidates (BIRC3, SLC9A5 & SMAD3) are more detected in "early tumors" than in 'late tumors'. Using this pipeline, we also identified NTRK3, HARS2 and PDGFRB as interesting candidates.

Source data are available in Figure appendix S3 source data.

Appendix Table 1 (Stats)

Raw data are available in Figures source data files.

Figure 3E

Mann Whitney test

pigmented	P value	<0,0001
vs	Exact or a	Exact
invasive	P value su	****

pigmented	P value	<0,0001
vs	Exact or a	Exact
NCSC	P value su	****

pigmented	P value	0,4954
vs,	Exact or a	Exact
SMC	P value su	ns

Figure 5C

One Way ANOVA

	P-value	
U *vs* NC	0.5822	ns
U *vs* T	0.0021	**
U *vs* M	<0.0001	***
NC *vs* T	0.0001	***
NC *vs* M	<0.0001	***
T *vs* M	0.1053	ns

Figure 5E

One Way ANOVA

	P-value	
CTR *vs* SMAD3	<0.0001	***
CTR *vs* BIRC3	0.1310	ns
CTR *vs* SLC9A5	0.0431	*

Figure 5H

One Way ANOVA

	P-value	
siCTR + BRAFi *vs* siSMAD3 #2 + BRAFi	0.1088	ns
siCTR + BRAFi *vs* siSMAD3 #1 + BRAFi	0.0058	**

Figure 5G

One Way ANOVA

	P-value	
siCTR + BRAFi *vs* siSMAD3 #2 + BRAFi	0.0367	*
siCTR + BRAFi *vs* siSMAD3 #1 + BRAFi	0.0020	**

Figure 5K

Bilateral Student test

	P-value	
SKMel28S - TGFb *vs* TGFb + SMAD3i	0.0261	*
SKMel28R - TGFb *vs* TGFb + SMAD3i	0.0309	*
M238S - TGFb *vs* TGFb + SMAD3i	0.0031	**
M238R - TGFb *vs* TGFb + SMAD3i	0.0463	*

Figure 6B

One Way ANOVA

	P-value	
DMSO *vs* TCDD	<0.0001	***
DMSO *vs* ITE	<0.0001	***

Figure 6C

One Way ANOVA

	P-value	
DMSO *vs* TCDD	0.0013	**
DMSO *vs* ITE	0.0008	***

Figure 6D

Bilateral Student test

	P-value	
TIPARP - DMSO *vs* CH-223191	0.0005	***
SMAD3 - DMSO *vs* CH-223191	<0.0001	***

Figure 7D

One Way ANOVA

	P-value	
U *vs* NC	0.0009	***
U *vs* T	<0.0001	***
U *vs* M	<0.0001	***
NC *vs* T	0.0043	**
NC *vs* M	<0.0001	***
T *vs* M	0.1985	ns

Figure 7F

Mann-Whitney test

	P-value	
Responder *vs* Non-Responder	0.0324	*

Figure 7H

Bilateral Student test

SKMel28R	P-value		Me1402	P-value	
CTR *vs* SMAD3	0.0004	***	CTR *vs* SMAD3	<0.0001	***
CTR *vs* NRP1			CTR *vs* NRP1		
CTR *vs* AXL	0.0044	**	CTR *vs* AXL	0.0013	**
CTR *vs* RUNX2	0.4861	ns	CTR *vs* RUNX2	0.1538	ns
CTR *vs* SLIT2	0.0640	ns	CTR *vs* SLIT2	0.0089	**
CTR *vs* JUNB	0.0052	**	CTR *vs* JUNB	0.0727	ns
CTR *vs* EGFR	0.0230	*	CTR *vs* EGFR	0.0199	*
CTR *vs* ITGB5	0.2967	ns	CTR *vs* ITGB5	0.0235	*
CTR *vs* AFAP1	0.0015	**	CTR *vs* AFAP1	0.0118	*
CTR *vs* MMP2	0.0063	**	CTR *vs* MMP2	0.1331	ns

Figure EV1 B

Bilateral Student test

	P-value	
DMSO *vs* Birinapant	0.0036	**

Figure EV1 C

Bilateral Student test

	P-value	
DMSO *vs* Birinapant	0.0011	**

Figure EV2 A

Mann-Whitney test

	P-value	
Rr *vs* Ra	0.0303	*

Figure EV3 B

Bilateral Student test

	P-value	
siCTR *vs* siSMAD3 #2	<0.0001	***
siCTR *vs* siSMAD3 #1	<0.0001	***

Figure EV3 D

One Way ANOVA

	P-value	
U *vs* NC	0.1259	ns
U *vs* T	0.0144	*
U *vs* M	0.0418	*
NC *vs* T	<0.0001	***
NC *vs* M	0.0002	***
T *vs* M	0.5037	ns

Figure EV3 F

One Way ANOVA

	P-value	
U *vs* NC	0.0065	**
U *vs* T	<0.0001	***
U *vs* M	<0.0001	***
NC *vs* T	<0.0001	***
NC *vs* M	<0.0001	***
T *vs* M	9135	ns

Figure EV3 H

Bilateral Student test

	P-value	
siCTR *vs* siBIRC3 #1	<0.0001	***
siCTR *vs* siBIRC3 #2	<0.0001	***

Figure EV3 I

Bilateral Student test

	P-value	
siCTR + BRAFi *vs* siEGFR#1 + BRAFi	0.2365	ns
siCTR + BRAFi *vs* siEGFR#2 + BRAFi	0.0359	*
siCTR + BRAFi *vs* siEGFR#3 + BRAFi	0.2019	ns
siCTR + BRAFi *vs* siIL6#1 + BRAFi	0.0584	ns
siCTR + BRAFi *vs* siIL6#2 + BRAFi	0.0483	*
siCTR + BRAFi *vs* siIL6#3 + BRAFi	0.0530	ns
siCTR + BRAFi *vs* AQP1#1 + BRAFi	0.1116	ns
siCTR + BRAFi *vs* AQP1#2 + BRAFi	0.0599	ns
siCTR + BRAFi *vs* AQP1#3 + BRAFi	0.0316	*

Figure EV3 J

Bilateral Student test

	P-value	
siCTR *vs* siEGFR#1	0.0004	***
siCTR *vs* siEGFR#2	0.0019	**
siCTR *vs* siEGFR#3	0.0014	**
siCTR *vs* siIL6#1		
siCTR *vs* siIL6#2	0.0010	**
siCTR *vs* siIL6#3		
siCTR *vs* siAQP1 #1		
siCTR *vs* siAQP1 #2		
siCTR *vs* siAQP1 #3		

Supplementary Methods

CRISPR-SAM Screen

Protocol adapted from Joung J. et al., Nat Protoc. 2017 (doi:10.1038).

<u>ACKNOWLEDGEMENTS</u>
The authors are grateful to Feng Zhang for providing the Human CRISPR 3-plasmid activation pooled library (SAM) (Addgene). Human CRISPR Activation Library (Pooled library) – Addgene #1000000057
This library consists of three components which are all provided:

1. A nucleolytically inactive Cas9-VP64 fusion (Addgene plasmid # 61425). Blasticidin resistance.
2. A gRNA incorporating two MS2 RNA aptamers at the tetraloop and stem-loop 2 (present in the libraries)
3. The MS2-P65-HSF1 plasmid which expresses the activation helper protein (Addgene plasmid #89308). Hygromycin resistance.

Generation of 501Mel 2+ cells (dCas9 and MS2-P65-HSF1)

501Mel cells (200,000 cells per well (MW6)) have been co-infected overnight with viral supernatants (multiplicity of infection, MOI~0.2) produced as detailed below:

D0: plating of **two T25** of HEK293T (DMEM + 10% FCS + 1% PS) at about 40% confluence.

D+1: at about 50-60% confluence, perform the transfection according to the following conditions:

For one T25: 3.2 µg lentiviral plasmid + 1.1µg pVSV-G + 2.1µg psPAX2 into 180µL of OptiMem (tube A) and 16 µL Lipofectamine 2000 into 180µL (tube B).

pool tube A into tube B: mix with your finger (10x) and incubate 15 min (RT).
Distribute on cells (containing 3 mL DMEM + 10% FCS + 1% PS).

5h after transfection, change the cell medium. Add the minimum volume (3 ml/flask T25).
D+3: collect the infectious medium and centrifuge it to discard cells and cell debris (1300rpm, 3min, RT). Carefully collect the supernatant and filter it on PVDF filter 0.45µm with a low pressure.

Cells have been exposed to infectious media in presence of polybrene (8µg/mL) overnight. The MOI is ~0.2. Conventionally, the viral titer obtained in our conditions is ~1 x10^6 TU/mL (TU for transduction unit).

After 5 days of antibiotic selection (Blasticidin (2 µg/mL) and Hygromycin B (200 µg/mL)), expression levels of dCas9 and MS2-p65-HSF1 were evaluated by RT-qPCR. Primer

sequences are available in Gautron et al., Nat. Comms 2020). These cells correspond to the "501Mel 2+ cells".

Amplification of the plasmid library lentiSAMv2 (cat. no. 1000000057)

I - Preparation of LB medium

LB Lennox to prepare plate:

References:
- Petri (Ø90mm h14,2mm) – VWR #391-0455.
- Squared Petri : NUNC for culture (245 x 245 x 25 mm; 500cm^2) – Dutscher #055064

Powder	gram	Ref.	Batch
Tryptone	10g	BD #211705	5027930
Yeast Extract	5g	BD #212750	3326451
NaCl	5g	Sigma #S7653	SZBE2100V
Agar	15g	BD #214010	5232955

Fill to 1 liter with H_2O milliQ. **Adjust pH to 7.0**.
Add a bar magnet.
Autoclave the medium: short cycle.
Allow the medium to return to a temperature of about 55 ° C in a water bath to avoid caking.
Add the antibiotic (Ampicillin 100µg/ml), homogenize on a magnetic plate (not by turning over to avoid bubbles).
Pour the petris. Wait for the setting in mass (several hours). Store at + 4 ° C. Protect plates with plastic food film and plastic bag. Check them one week later for sterility.

LB Miller to collect the colonies on the boxes:

Powder	gram	Ref.	Batch
Tryptone	10g	BD #211705	5027930
Yeast Extract	5g	BD #212750	3326451
NaCl	10g	Sigma #S7653	SZBE2100V

Fill to 1 liter with H_2O milliQ. **Adjust pH to 7.0.**
Autoclave the medium: short cycle.
Allow the medium to cool, and store at + 4 ° C.

II –Bank Amplification

Amplification of the SAM library is performed by electroporation of electrocompetent bacteria.

References:

- Electroporator : Bio-Rad Micro Pulser (Cat. #165-2100)
- Electroporation vats : Bio-Rad Gene pulser cuvette 0.1cm 50 pK (Cat. #1652086)
- Bacteria : Lucigen E cloni. 10G Elite (Cat. #60051-2) ; batch n°11514
- Lucigen pUC19 DNA -10pg/µL (Pt#F92078-1) ; batch n°11158
- Lucigen Recovery medium (Cat. #80026-1) ; batch n°11390
- Human CRISPR Activation Library (Pooled library) – Addgene #1000000057
- Maxiprep : NucleoBond Xtra Maxi (Macherey-Nagel ; ref : 740414.10)
- Conical centrifugation pots 250mL (Corning ; ref : 430776)

NOTE: Before carrying out the amplification of the library, the transformation efficiency must be tested using the equipment listed above.

- **Electroporation trial:**

25 µL of electrocompetent bacteria were transformed with 10 pg of pUC19 (available with bacteria).
Electroporation was performed with the "bacteria" ("standard") mode of the electroporator.
After the electric shock, the bacteria were taken a few minutes later (the time to go into bacteriology room) by 2mL of "recovery medium".
Isolation was performed on a Ø90mm petri plate (LB Lennox) from 10µL of the diluted bacterial solution above.

After an overnight incubation at 37 ° C (box turned), 100 colonies were counted on the plate.
100 colonies for 10µl from 2mL (recovery medium). So, for 10 pg used, 20,000 colonies were obtained. Thus, for 1µg, we would obtained 2 x10^9cfu / µg. Efficiency = 2x10^9 cfu / µg.
Minimum recommended efficiency for the amplification of the library = 1 x10^9cfu / µg.

Note: Size of pUC19(2,7kb) is different to lenti sgRNA MS2 (9.9kb). So, the efficiency will be less efficient with the lentiviral plasmid.

- **Plasmid library Amplification:**

To amplify the plasmid library, it is necessary to have poured (LB Lennox) at least 8 squared boxes NUNC (245 * 245 * 25 mm) and 1 petri dish Ø90mm.
Note: It is advisable to prepare much more to overcome various problems (agar damaged, etc.). It is therefore advisable to prepare at least 12 squared boxes and 3 boxes of petri dishes. Before beginning the transformation, incubate these dishes, the lid on top, in the incubator at 37 ° C for about 2 hours. Keep them at 37°C during the transformation.

The following steps are performed around flame (bunsen burner) on a bench near the electroporator.

1µL of the bank (eq 50ng) is added on 25µL of bacteria. The mix is gently homogenized by 2 go/return movements into the tip of the pipette 200 before transfer into the electroporation cell.
Note: Be careful, do not make a bubble when you put the mix in the tank! Electroporation was performed with the "bacteria" ("standard") mode of the electroporator. Then, as soon as possible, 975µL of "sterile recovery medium" is gently added to the tank, **without going back and forth** but trying to create a slight current in the bottom of the tank during the addition. Then transfer everything into a sterile 2mL Eppendorf that is placed on ice.

This step is carried out 8 times (1 µl of plasmid library on 8 different tubes of bacteria). To facilitate the handling, it is easier to work with somebody: one who deals with the "mix" then transfer into the tank and at the end of the addition of the medium, and another one who performs the electroporation. This allows to be faster.

Once the 8 repetitions are done, add 1mL of "recovery medium" in all the tubes.
Note: Also prepare a 2mL Eppendorf tube with 1mL of "recovery medium" for calculating the transformation efficiency.

Climb bacteriology room. Incubate the 8 tubes at 37°C for **1 hour.**

Under laminar flow: Pool the 8 tubes (16mL in total) in a sterile 50mL Falcon.

Note: Take 20µL that is added to the tube containing 1mL of "recovery medium" prepared previously. Spread 100µL of this solution on the Ø90mm petri dish and incubate bottom lid at 37°C overnight. It will be used to calculate the transformation efficiency of the current experiment.

Always Under laminar flow, **spread 2 mL of the bacterial pool** (Falcon 50mL) **on each square box** using a "blue rake". Let "drink" these boxes at 37 ° C, cover up, for ~1h before turning and incubate on the night.

Before recovering the bacteria from the squared boxes, it is necessary to validate the efficiency of transformation thanks to the petri dish prepared previously.

On this box, we got ~300 colonies on 1/8th of the box. That is about 2400 colonies in total. So, 2400 colonies for 100µL. These 100µL correspond to 1/10th of the 2mL solution containing 20µL of bacteria from the 16mL spread in total. So total colony number = 2400 * 10 * 8000 = 1.92 x10^8colonies.
It is recommended a minimum of 7 x10^7colonies (eq 100X colonies per guide).

NB: think about weighing the conical pots that will be used to pack the bacteria.

After removing the plates from the incubator, they are scraped 2 by 2. Indeed, to recover the bacteria, 10mL of LB Miller medium are added to each box, which are scraped with a small cell scraper. The detached bacteria are then transferred to a conical pot for centrifugation. A second phase of scraping is carried out on each box with 10ml of medium, and the bacteria recovered in this same plot. Two pots are used: 4 plates / pot.

Once the assembly is complete, the bacteria are centrifuged for 30 min - 3000 rpm - 4 ° C. The supernatant is carefully removed by inversion and the pots and the pellets are

weighed. **Otherwise the LB distorts the weighing.** Pot n ° 1: 33,87g - Pot n ° 2: 33,71g. Therefore, weight of the pellets: No. 1 pellet = 4.22 g - No. 2 pellet = 3.86 g**.**

The plasmids of the library are purified using a kit of Maxiprep, at the rate of **a maxiprep for about 0.75g of bacteria. Here we used 11 maxipreps**. Plasmids were checked on BET gel and nanodrop.
Aliquots were stored at -80°C.

From Plasmids to Lentivirus

I –Lentivirus Production

D0: prepare **twelve T225** of HEK293T cells (DMEM + 10% FCS + 1% PS) at about 40% confluence. (6 full flasks T225 full to generate 12 **flasks T225 containing** 30×10^6 HEK293T cells).

D+1: at about 50-60% confluence, perform the transfection according to the following conditions:

For one T225: 10µg sgRNA library + 10µg pVSV-G + 15µg psPAX2 and 90µL Lipofectamine 2000. So, for each virus production: 120µg VSV-G and 180µg PAX2 and 120µg library)

For **12**flasks T225:

Tube A: pVSVG	1172 ng/µL	120 µg	102 µL
Tube A: psPAX2	934 ng/µL	180 µg	193 µL
Tube A: SAM bank	2478 ng/µL	120 µg	48µL
Tube A: OPTI-MEM	16,2 mL		
Tube B : Lipo2000	1080 µL		
Tube B: OPTI-MEM	16,2 mL		

Mix tube A into tube B: mix with your fingers (10x) and incubate 15 min (RT).
Distribute 2.8 mL per T225 (containing 25mL DMEM + 10% FCS + 1% PS).

5h after transfection, change the cell medium. Add the minimum volume (25 mL/flask).
D+3: Collect the infectious media and centrifuge them (1300rpm, 3min, RT) in falcon 50mL.
Collect the supernatants (25mL) and filter them **with a low pressure** (50mL syringe) on a **0.45µm PVDF** filter (**NOT cellulose filter**). **ONE filter per T225** (25mL).
Pool supernatants and homogenize gently. **To use immediately (about 300mL). If you store this viral supernatants, ~50% of virus is destroyed. Keep in mind for MOI calculation.**

NOTE : D+2: 4 x T225 containing 10×10^6 501Mel2+ were prepared cells (RPMI + 10% FCS + 1% PS). Six petris must also be prepared (Ø100mm) with 1×10^6 of 501Mel2+ cells/petri for the MOI calculation.

• **<u>The Multiplicity of infection sought is 0.2</u>**. From the observations of the first experiment, we used a volume of 40mL of virus (0.22x10^6 TU/mL) for 40M of cells. (40mL x 0.22x10^6 TU/mL = 8.8 x10^6 TU for 40x10^6 cells. MOI=0.22. **It is important to keep in mind that at an MOI 0.3 or less, greater than 95% of infected cells are predicted to have a single integration and is therefore recommended for pooled screening.**
8.8 x10^6 TU and the library contains 70290 sgRNA (guides). In this case we will obtain ~125 infected cells expressing the same guide). So, 10mL of viral supernatant have been added per flask with polybrene (4µg/µL final). Infection overnight.

II –MOI Calculation (post-infection)

The infection should be made at a MOI of 0.2. To test the MOI, carry out an infection of 501Mel2+ in P100 (1M of 25% confluent cells) with 6 different dilutions of lentivirus :100µL/petri (100th, 200th, 500th, 1000th, 2000th and without virus).
First prepare 500µL of viral supernatant diluted 1: 100 (5 µL + 495 µL medium)

Dilution	**Infectious medium**	Volume medium
1/100	100 µL	/
1/200	50 µL	50 µL
1/500	20 µL	80 µL
1/1000	10 µL	90 µL
1/2000	5 µL	95 µL

If you have time, it is interesting to test additional dilution 1/50, 1/20, etc.

After 3 to 6 days of antibiotic selection (zeocin at 600 µg/mL). Count surviving colonies after staining with methylene blue (Gilot D. et al., Nat Cell Biol 2017). **NO colony in petri without virus (negative control) at the end of antibiotic selection.**
For example, 110 colonies were counted in the 1/200 dilution, which indicates 22,000 TU/100µL of this dilution (1/200). Thus, our viral supernatant contained 0.22x10^6 TU/mL. Similar results were obtained with the other dilutions (41 for 1/500 et 20 for 1/1000). We estimated the titer ~0.2x10^6 TU/mL.

<u>III–Cell Selection</u>

D+1 (24 h post infection): count the cells and split them in several T225 at the rate of 7.5 x10^6 cells per T225 (low density seeding) to obtain a better and faster antibiotic selection. Knowing that we seeded 10M by T225 two days ago, we consider that we will obtain ~25M cell by T225, so 100 x10^6 cells per condition. **Provide 14 T225/condition.**

3h after the plating (cells are adherent), **apply the antibiotic selection** (here : zeocin (300 µg/mL then 600 µg/mL).

Day	Amount	Volume (medium)	Volume Zeocin	
D+1	300µg/mL	980mL	2940 µL	

D+3	600µg/mL	980mL	5880 µL	
D+5 =end of selection	0µg/mL	980mL	0	If selection is not complete, expose cells to 600µg/mL
Total		2940 mL	**8820** µL	Or **14700**µL

D+7: Split the cells in T225 at ~6-7 M cell/T225.
As soon as possible, make a pellet of at least 36 x10^6 cells and make freezing tubes with the same amount.
For the screen with this library, it is necessary to work with at least 500 cells per sgRNA (500x70,290 = 35,145,000 cells). It is therefore necessary to keep at least 36x10^6 cells for the experiments. We used 40x10^6 cells for each experimental condition.

Note: In our manuscript (Fig.1, Gautron A. et al. 2020), we estimated the coverage (>95%), the sgRNA distribution (mean 550) and the correlation between replicates (Control (DMSO): r=0.86 and for BRAFi: r=0.95).

From Cell Bank to Resistant Cells

Note: If you start from already infected and frozen cells, **you have to do a centrifugation to eliminate the medium + DMSO, otherwise the cells do not adhere well**.

D+7: Resuspend the cells and separate them in different conditions according to the treatments. Here DMSO and BRAFi. Therefore, 40x10^6 cells * 3 = 120 x10^6 cells. Cells were split to control (DMSO= solvent of BRAFi) and drug treatment arms (BRAFi; PLX4032 (2µM, Selleckchem) or Paradox Breaker (PLX8394, 2µM, MedChemExpress)). **During the treatment (14 days), media were renewed every two days** in order to eliminate dead cells and to ensure a potent BRAFi pressure.

D+21: After 14 days of treatment, make a pellet of each condition. Cells were pelleted by centrifugation, resuspended in PBS, and frozen promptly for genomic DNA isolation to identify sgRNA sequences. Pellets could be stored (-80°C).

From Cell Pellets to Guide Identification

For this part, we used the protocol published by Julia Joung (Nature Protoc, 2017).
Genomic DNAs from cell pellets and tumors (>400mg) were extracted using the Zymo Research Quick-gDNA MidiPrep according to the manufacturer's protocol. PCR amplifications and quality controls have been done as described by Zhang lab (Joung et al., 2017).

<u>I –Library</u>

- Genomic DNA was extracted using the Zymo Quick-gDNA midi kit (Zymo Research). One pellet split on 4 columns (max 10x10^6 cells/column). We obtained at least 250µg/pellet.

- DNA Quantification has been made with Nanodrop and BioAnalyzer.
- PCR have been performed using the NEBNextHigh Fidelity 2X Master Mix. 32 cycles/single-step reaction.

For one sample, library is made using 10 forward primers (diversity) for one Reverse primer (Reverse primer used for multiplexing). Eight PCR per primer couple. So, we generate 80 wells per sample.

2.25µg/well (PCR).

For another sample, use the same 10 forward primers but change the Reverse primer (cf supplementary Table Konermann et al., Nature 2015).

Purify pooled PCR products using Zymo spin V with reservoir

PCR products have been separated on agarose Gel (2%) to eliminate nucleotides and non-desired amplicons. **Note: check the bubble product (generated by PCR) with the BioAnalyser and on agarose gel. Only one PCR product should be detected and purified.**

Illustration: BioAnalyser profile of PCR-products before gel migration. Expected size (bank)~280bp. Bubble product >400bp.

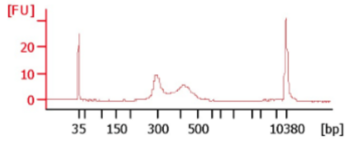

PCR products have been Gel extracted using Zymoclean Gel DNA Recovery Kit - Uncapped columns

The library is sequenced on HiSeq (35 million reads passing filter per library).

II –Sequencing

Sequencing was performed by the Human & Environmental Genomics core facility of Rennes on a HiSeq 1500 (Rapid SBS kit v2 1x100 cycles, Illumina). Base Calling was performed with Illumina's CASAVA pipeline (Version 1.8).

III –SgRNA Enrichment Analysis

Data processing was conducted using the MAGeCK v0.5.6 software. Briefly, read counts from different samples are first median-normalized to adjust for the effect of library sizes and read count distributions (mageck count with option: --norm-method median). Then, in an approach similar to those used for differential RNA-Seq analysis, the variance of read counts is estimated by sharing information across features and a negative binomial model is used to test whether sgRNA abundance differs significantly between the treatment conditions and the DMSO control. Positively or negatively selected sgRNA are ranked according to adjusted P-values (false discovery rate) and gene log fold changes computed with the modified robust ranking aggregation algorithm implemented in MAGeCK (mageck test with options: --norm-method median, --gene-lfc-method alphamedian, --adjus

SUPPLEMENTARY REFERENCES

Marin-Bejar O, Rogiers A, Dewaele M, Femel J, Karras P, Pozniak J, Bervoets G, Raemdonck N Van, Pedri D, Swings T, *et al* (2020) A neural crest stem cell-like state drives nongenetic resistance to targeted therapy in melanoma. *bioRxiv*: 2020.12.15.422929

Ohanna M, Giuliano S, Bonet C, Imbert V, Hofman V, Zangari J, Bille K, Robert C, Bressac-de Paillerets B, Hofman P, *et al* (2011) Senescent cells develop a PARP-1 and nuclear factor- B-associated secretome (PNAS). *Genes Dev* 25: 1245–1261
