# Supplementary material for: CRISPR screens identify tumor‐promoting genes conferring melanoma cell plasticity and resistance
Source: EMBO Mol Med. 2021 Mar 16;13(5):e13466. doi: 10.15252/emmm.202013466 (PMC8103100; doi:10.15252/emmm.202013466)
Supplement: Supplementary file 2 — Expanded View Figures PDF [file EMMM-13-e13466-s011.pdf]

Expanded View Figures

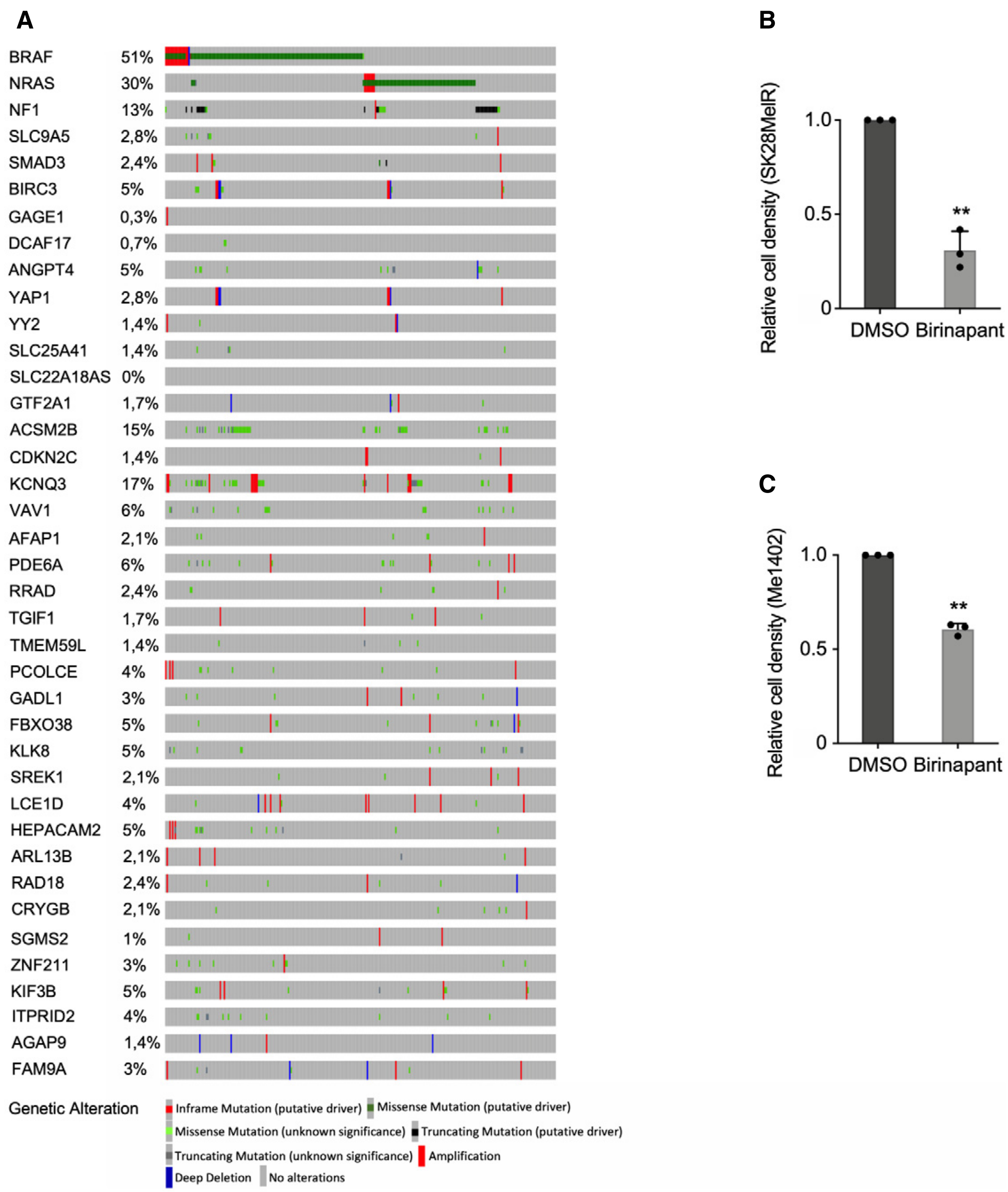

Figure EV1. Tumor-promoting genes are not frequently altered in cutaneous melanoma. Related to Fig 1.

A Genomic alterations and 36 tumor-promoting genes identified in Fig 1. *BRAF*, *NRAS*, and *NF1* have been added to the list of tumor-promoting genes according to melanoma classification (Akbani *et al*, 2015). Analyses were performed using the webtool available at www.cbioportal.org.

B Birinapant, an inhibitor of BIRC proteins (BIRC3 and BIRC2), decreased cell density (SKMel28R cells, Birinapant 100 nM, 96 h).

C Birinapant, an inhibitor of BIRC proteins (BIRC3 and BIRC2), decreased cell density (Me1402 cells, Birinapant 100 nM, 96 h).

Data information: Each histogram represents the mean ± s.d. from three independent experiments. Bilateral Student test (with non-equivalent variances) $^{**}P < 0.01$. Source data are available online for this figure.

© 2021 The Authors

Figure EV2. BRAFi-resistance genes are associated with invasive phenotype and resistance to BRAFi *in vitro* and *in vivo*. Related to Fig 3.

A *BIRC3* expression levels (fold change) in melanoma Rr and Ra cell lines. The fold change has been calculated by comparing *BIRC3* expression levels in resistant/parental cell lines (Song *et al*, 2017). Two groups have been made: Rr (n = 6) and Ra (n = 5) cell lines; Ra for resistance and MAPK reactivation and Rr for resistance and MAPK redundant. BRAFi resistance in Ra cell lines is due to additional genetic alterations such as BRAF splicing events for M395 SDR. BRAFi resistance in Rr cell lines is due to gene expression reprogramming. Two-tailed Mann–Whitney test, P = 0.0303. Vertical bars correspond to the medians.

B Schematic representation of the workflow. As described in the original paper (Shaffer *et al*, 2017), fresh melanoma cells were obtained by patient tumor dissociation. Cells mix was treated with BRAFi. Cell sorting (EGFR positive and/or negative) was performed at two time points: before treatment (no drug) and after 4 weeks of treatment. Cells “mix” corresponds to the unsorted population. The EGFR-positive cells (exposed to BRAFi) are able to produce colonies.

C Heat map depicting mRNA expression of BRAFi-resistance genes, using an RNA-Seq dataset obtained from BRAFi-treated melanoma cells (Shaffer *et al*, 2017). Scale corresponds to Z scores.

D Heat map illustrating the expression levels of BRAFi-resistance genes in invasive (Inv.) and proliferative melanoma cell lines (n = 2 and n = 9, respectively) (Verfaillie *et al*, 2015). Scale corresponds to Z scores.

E Genomic alterations in 18 BRAFi-resistance genes identified in Fig 3 (from www.cbioportal.org). *BRAF*, *NRAS*, and *NF1* have been added according to melanoma classification (Akbani *et al*, 2015).

F *SMAD3* expression levels (RNA Seq V2 ($\log_2$) in the TCGA dataset. Picture was downloaded from www.cbioportal.org. Arrow indicates cutaneous melanoma.

G *TYRP1* expression levels (RNA Seq V2 ($\log_2$) in the TCGA data set. Pictures were downloaded from www.cbioportal.org. Arrow indicates cutaneous melanoma. *TYRP1* is highly expressed in drug-naive tumors; cutaneous and uveal melanoma in contrast to *SMAD3*.

H *SMAD3* expression and cell types (https://www.proteinatlas.org/ENSG00000166949-SMAD3/celltype). The consensus normalized expression (NX) value for *SMAD3* and organ/tissue represents the maximum NX value in the three data sources (HPA, GTEx, and FANTOM5). Black arrow highlights the melanocytes.

Source data are available online for this figure.

© 2021 The Authors

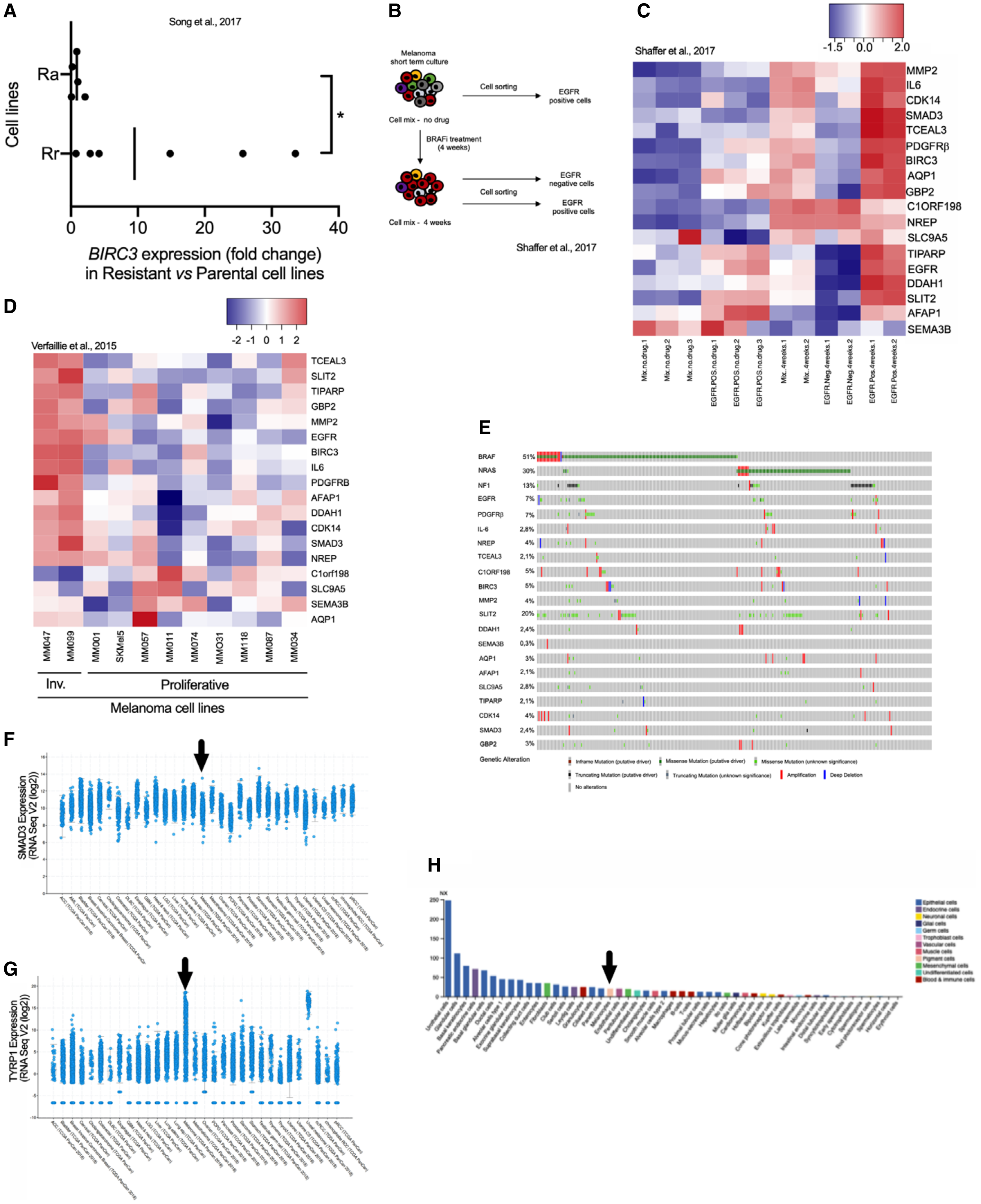
A
Song et al., 2017
Ra
Rr
Cell lines
*
0
10
20
30
40
BIRC3 expression (fold change)
in Resistant vs Parental cell lines
B
Melanoma
short term culture
Cell mix - no drug
Cell sorting
EGFR
positive cells
BRAFi treatment
(4 weeks)
Cell mix - 4 weeks
EGFR
negative cells
Cell sorting
EGFR
positive cells
Shaffer et al., 2017
C
Shaffer et al., 2017
-1.5
0.0
2.0
MMP2
IL6
CDK14
SMAD3
TCEAL3
PDGFRβ
BIRC3
AQP1
GBP2
C1ORF198
NREP
SLC9A5
TIPARP
EGFR
DDAH1
SLIT2
AFAP1
SEMA3B
Mix.no.drug.1
Mix.no.drug.2
Mix.no.drug.3
EGFR.POS.no.drug.1
EGFR.POS.no.drug.2
EGFR.POS.no.drug.3
Mix..4weeks.1
Mix..4weeks.2
EGFR.Neg.4weeks.1
EGFR.Neg.4weeks.2
EGFR.Pos.4weeks.1
EGFR.Pos.4weeks.2
D
Verfaillie et al., 2015
-2
-1
0
1
2
TCEAL3
SLIT2
TIPARP
GBP2
MMP2
EGFR
BIRC3
IL6
PDGFRB
AFAP1
DDAH1
CDK14
SMAD3
NREP
C1orf198
SLC9A5
SEMA3B
AQP1
MM047
MM099
MM001
SKMel5
MM057
MM011
MM074
MMO31
MM118
MM087
MM034
Inv.
Proliferative
Melanoma cell lines
E
BRAF 51%
NRAS 30%
NF1 13%
EGFR 7%
PDGFRβ 7%
IL-6 2,8%
NREP 4%
TCEAL3 2,1%
C1ORF198 5%
BIRC3 5%
MMP2 4%
SLIT2 20%
DDAH1 2,4%
SEMA3B 0,3%
AQP1 3%
AFAP1 2,1%
SLC9A5 2,8%
TIPARP 2,1%
CDK14 4%
SMAD3 2,4%
GBP2 3%
Genetic Alteration
Inframe Mutation (putative driver)
Missense Mutation (putative driver)
Missense Mutation (unknown significance)
Truncating Mutation (putative driver)
Truncating Mutation (unknown significance)
Amplification
Deep Deletion
No alterations
F
SMAD3 Expression
(RNA Seq V2 (log2))
G
TYRP1 Expression
(RNA Seq V2 (log2))
H
NX
250
200
150
100
50
0

Figure EV2.

Figure EV3. *BIRC3* and *EGFR* are potent BRAFi-resistance genes. Related to Fig 5.

- A Pictures illustrating the ratio (invasive diameter/spheroid diameter; E/S) used in Fig 5E (Invasion assay, melanoma spheroids). Two cell lines are illustrated: CTR cells correspond to 501Mel cells expressing dCas9 and HSF1-p65-MS2 (named here 501Mel 2+) and the SMAD3 cells (overexpressing SMAD3).
- B SMAD3 Knock-down efficiency of two siRNAs targeting *SMAD3* in SKMel28R (Fig 5G). SMAD3 protein expression levels have been quantified in three independent experiments by Western blots. Expression levels have been compared to HSC70. Values obtained with the control siRNA (CTR) have been set at 1. Bilateral Student test (with non-equivalent variances) ***$P < 0.001$.
- C PCA analysis of *BIRC3* expression in melanoma cell lines in function of their dedifferentiation states (generated by the webtool http://systems.crump.ucla.edu/dediff/index.php). Scale: red color corresponds to a high *BIRC3* expression level (unit of the scale: Log_2 FPKM).
- D *BIRC3* expression in these four subtypes of melanoma cells (undifferentiated = 10; neural crest-like = 14; transitory = 12; melanocytic = 17) (Tsoi *et al*, 2018). Error bars represents the mean ± s.d. Multiple comparisons have been done using ordinary one-way ANOVA, *$P < 0.05$, ***$P < 0.001$.
- E PCA analysis of *EGFR* expression in melanoma cell lines in function of their dedifferentiation states (unit of the scale: Log_2 FPKM).
- F *EGFR* expression in these four subtypes of melanoma cells (undifferentiated = 10; neural crest-like = 14; transitory = 12; melanocytic = 17). Error bars represents the mean ± s.d. Multiple comparisons have been done using ordinary one-way ANOVA, **$P < 0.01$, ***$P < 0.001$.
- G *BIRC3* depletion (siRNA#1 & #2) reduced cell density and restored BRAFi (vemurafenib) effect on BRAFi-resistant cells (SKMel28R). CTR for non-targeting siRNA. Vem for the BRAFi vemurafenib and DMSO for dimethylsulfoxide (solvent of Vem). $n = 2$ biologically independent experiments. Each histogram represents the mean ± s.d.
- H *BIRC3* Knock-down efficiency of two siRNAs targeting *BIRC3* in SKMel28R (Fig EV3G). *BIRC3* mRNA expression levels have been quantified in three independent experiments by RT–qPCR. Values obtained with the control siRNA (CTR) have been set at 1. Each histogram represents the mean ± s.d. $n = 3$ biologically independent experiments. Bilateral Student test (with non-equivalent variances) ***$P < 0.001$.
- I Knock-down of *EGFR, IL6, or AQP1* (siRNA#1, #2, and #3) restored BRAFi (vemurafenib) effect on BRAFi-resistant cells (SKMel28R). $n = 3$ biologically independent experiments are presented. Each histogram represents the mean ± s.d.. Bilateral Student test (with non-equivalent variances) *$P < 0.05$.
- J Knock-down efficiency of siRNAs targeting *EGFR, IL6, or AQP1* in SKMel28R (Fig EV3I). mRNA expression levels have been quantified in two or three independent experiments by RT–qPCR. Each histogram represents the mean ± s.d. $n = 3$ biologically independent experiments for siCTR, siEGFR#1,2,4, IL6#3, and $n = 2$ for the other siRNAs. Values obtained with the control siRNA (CTR) have been set at 1. Bilateral Student test (with non-equivalent variances) **$P < 0.01$, ***$P < 0.001$
- K Efficiency of BRAFi (Vem) and MEKi (Cobi) to inhibit the MAPK signaling pathway (P-ERK) (as used in Fig 5O). Values obtained with the solvent (CTR, here DMSO) have been set at 100%.

Source data are available online for this figure.

 © 2021 The Authors

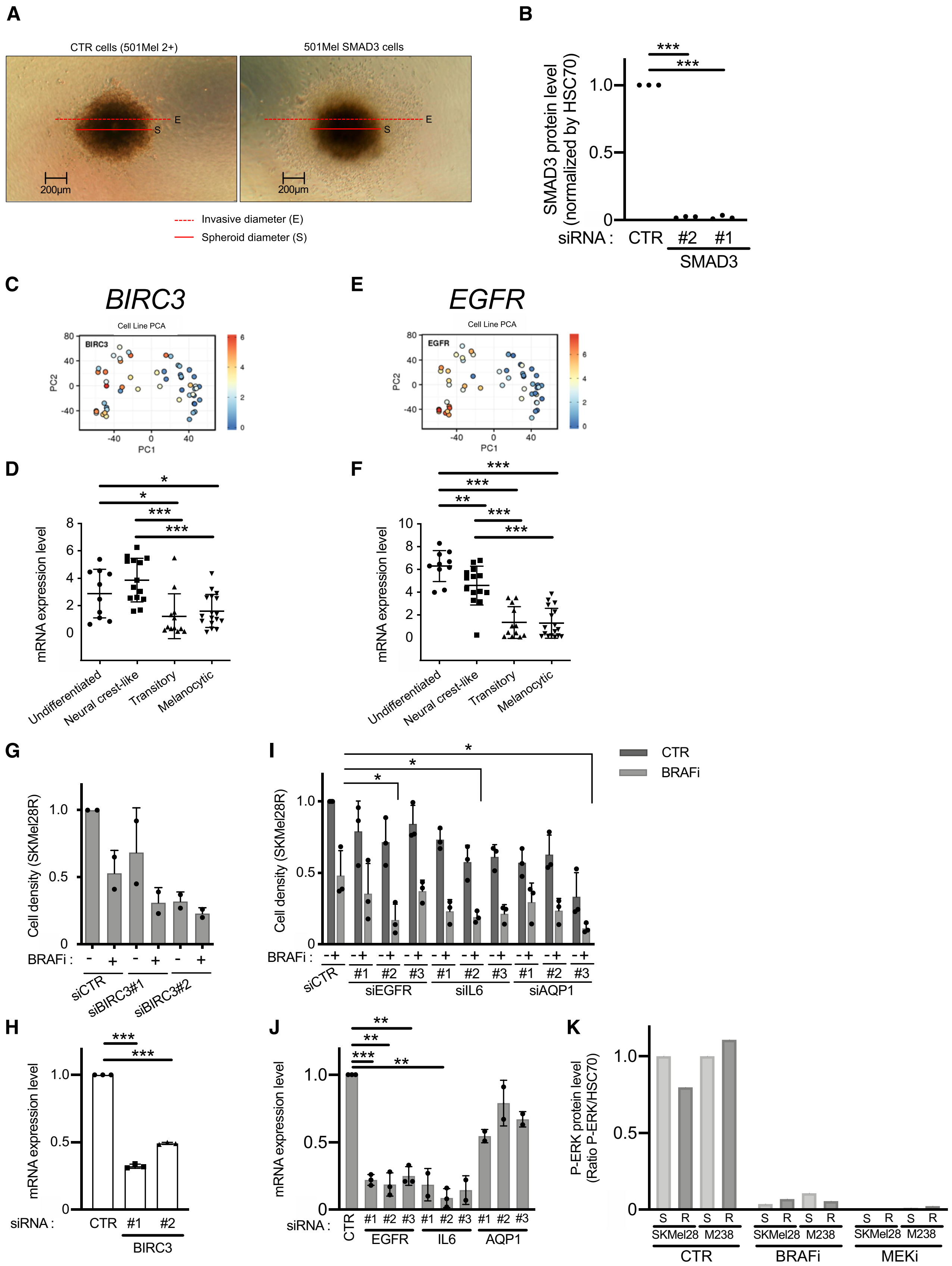
A
CTR cells (501Mel 2+)
501Mel SMAD3 cells
E
S
200µm
Invasive diameter (E)
Spheroid diameter (S)
B
SMAD3 protein level (normalized by HSC70)
siRNA : CTR #2 #1
SMAD3
C
BIRC3
Cell Line PCA
E
EGFR
Cell Line PCA
PC1
PC2
D
F
mRNA expression level
Undifferentiated
Neural crest-like
Transitory
Melanocytic
G
Cell density (SKMel28R)
BRAFi :
siCTR
siBIRC3#1
siBIRC3#2
I
siEGFR
siIL6
siAQP1
CTR
BRAFi
H
siRNA : CTR #1 #2
BIRC3
J
EGFR
IL6
AQP1
K
P-ERK protein level (Ratio P-ERK/HSC70)
SKMel28 M238
CTR
BRAFi
MEKi

Figure EV3.

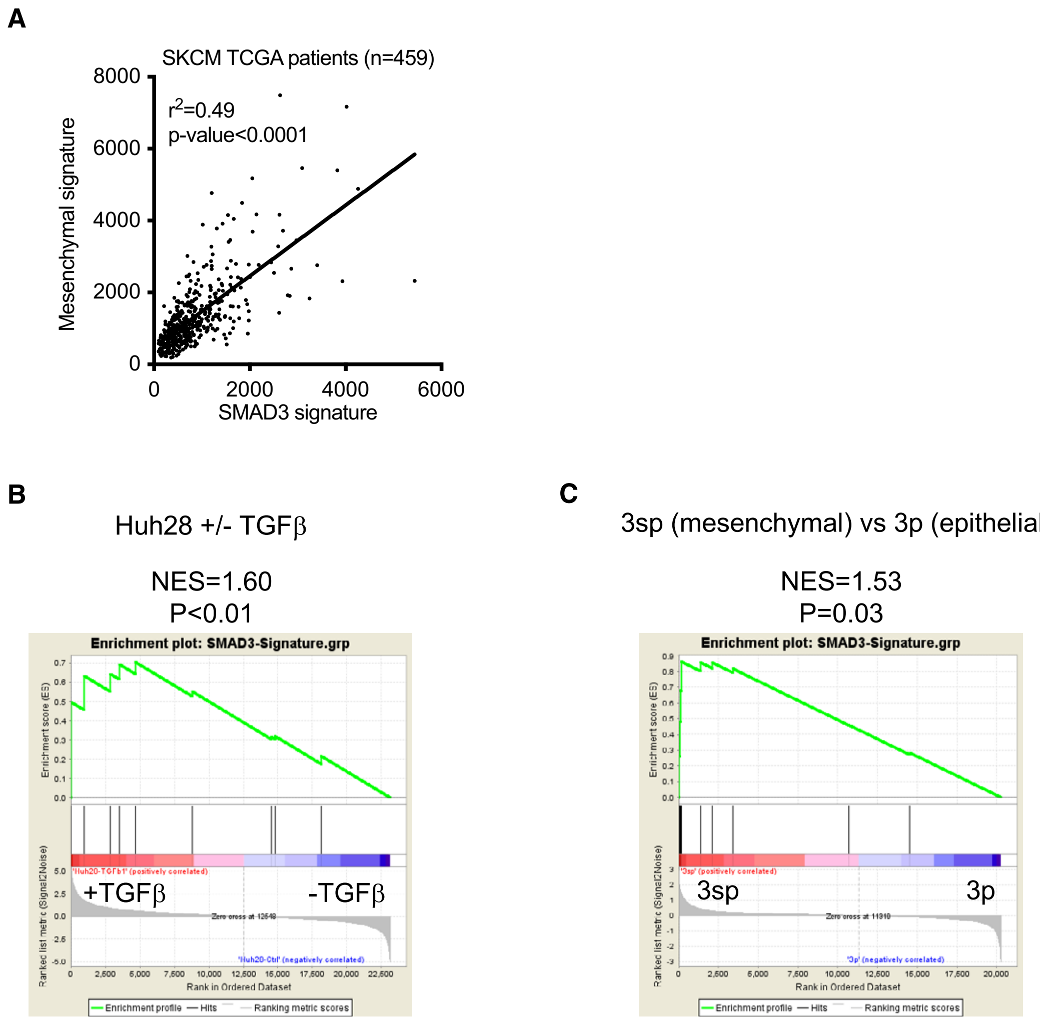

Figure EV4. SMAD3 signature is associated with mesenchymal phenotype in liver cancer cell lines. Related to Fig 7.

A Plot showing correlation between SMAD3-signature and mesenchymal signature (Mak *et al*, 2016) in the TCGA dataset (SKCM). Pearson correlation test: $P < 0.0001$

B Gene Set Enrichment Analysis (GSEA) of the SMAD3 signature in TGF-β treated *versus* non-treated Huh-28 cholangiocarcinoma cells (Merdrignac *et al*, 2018).

C Gene Set Enrichment Analysis of the SMAD3 signature in hepatoma cell lines : mesenchymal (3sp cells) *versus* epithelial (3p cells) (van Zijl *et al*, 2011).

 © 2021 The Authors

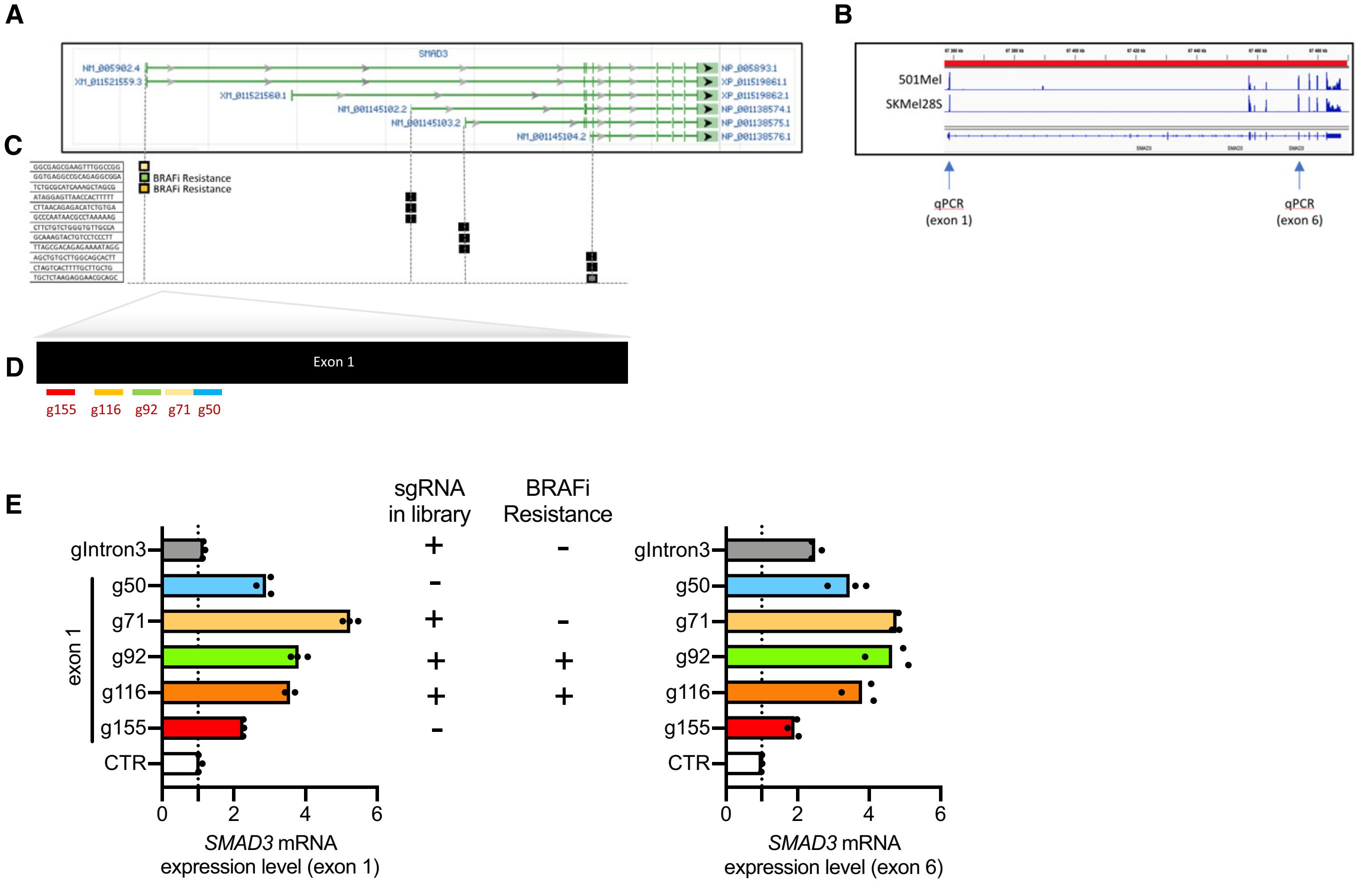

Figure EV5. sgRNA targeting four isoforms of *SMAD3* and BRAFi resistance.

- A *SMAD3* mRNA isoforms according to NCBI. The longest isoform NM_005902.4 encodes SMAD3 protein (NP_005893.1). Three shorter isoforms of *SMAD3* mRNA encode shorter SMAD3 proteins.
- B Snapshot of *SMAD3* mRNA expression in 501Mel and SKMel28S cells using IGV tool. Note that exon 1 is detectable in these melanoma cell lines. Arrows indicate the binding sites of primers for RT–qPCR (exon 1 or 6 for NM_005902.4).
- C Scheme to illustrate the binding site of sgRNAs targeting *SMAD3* gene. These 12 sgRNAs are found in the CRISPR-SAM library (Konermann *et al*, 2015). Three sgRNAs per isoform. Only two sgRNA targeting the longest isoform of *SMAD3* are associated with BRAFi resistance (Fig 2).
- D Ability of six sgRNAs to transactivate *SMAD3* expression have been compared. Among the five sgRNAs targeting the first exon, three are found in the library (g71, g92, and g116). We designed g50 and g155 to evaluate the impact of sgRNA position on exon 1. sgRNA gIntron3 has been designed as a control (unable to promote the longest *SMAD3* isoform but efficient to transactivate the smallest isoform). These two isoforms contain the exon 6 used for RT–qPCR.
- E *SMAD3* quantification by RT–qPCR (exon 1 or 6) in HEK293 cells transiently transfected with the three plasmids required for the CRISPR-SAM as previously described (Konermann *et al*, 2015). CTR for empty plasmid (no sgRNA cloned into lenti sgRNA(MS2)_zeo backbone). RT–qPCR data are representative of two independent experiments. Source data are available in Fig EV5 source data. Each histogram represents the mean of three technical replicates from one representative experiment. No statistical analyses were thus performed. Dotted line highlights the value of 1 (corresponding to the effect obtained by the control sgRNA).

Source data are available online for this figure.
