# Supplementary figures and images for: CRISPR screens identify tumor‐promoting genes conferring melanoma cell plasticity and resistance
Source: EMBO Mol Med. 2021 Mar 16;13(5):e13466. doi: 10.15252/emmm.202013466 (PMC8103100; doi:10.15252/emmm.202013466)

Fig. 1I

SMAD3

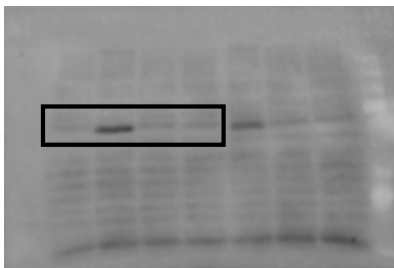

HSC70

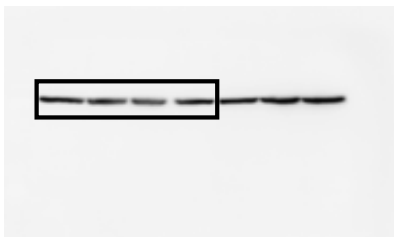

Source data file 2: Western-blot

Supplement: Supplementary file 13 — Source Data for Figure 1 [file EMMM-13-e13466-s019.zip › Source_data_file_2.pdf]

Fig. 5I

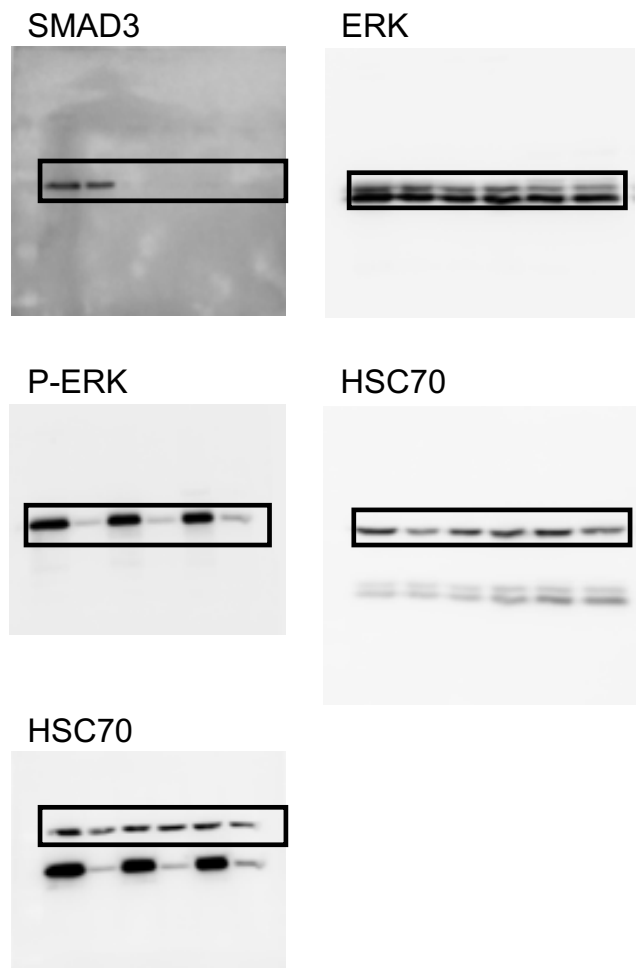

Fig. 5J

P-SMAD3

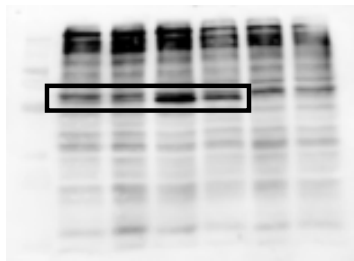

SMAD3

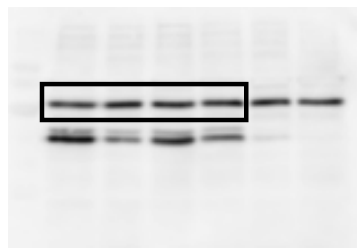

HSC70

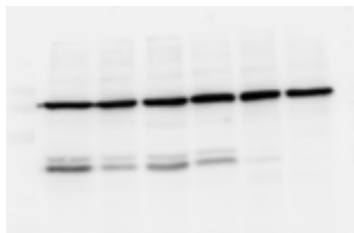

Fig. 5L

SMAD3

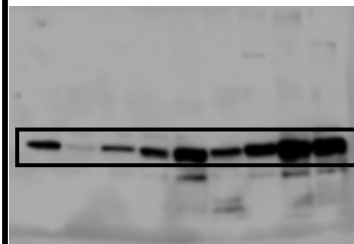

BIRC3

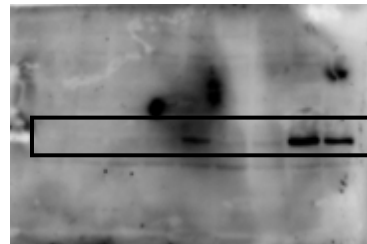

P-SMAD3

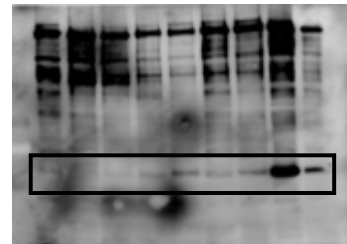

HSC70

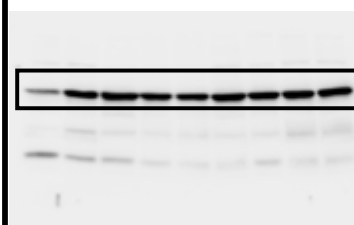

HSC70

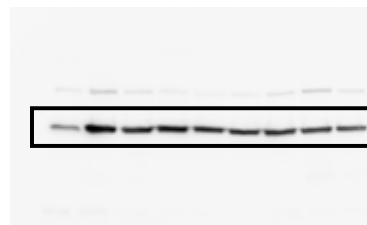

HSC70

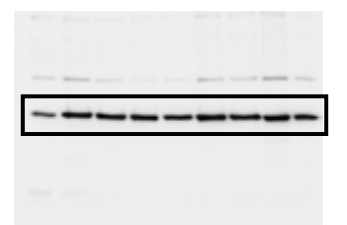

Supplement: Supplementary file 17 — Source Data for Figure 5 [file EMMM-13-e13466-s006.zip › Source_data_file_3.pdf]
